# Genetic Variant in Nicotinic Receptor α4-Subunit Causes Sleep-Related Hyperkinetic Epilepsy via Increased Channel Opening

**DOI:** 10.3390/ijms232012124

**Published:** 2022-10-12

**Authors:** Simone Mazzaferro, Deborah J. Msekela, Edward C. Cooper, Atul Maheshwari, Steven M. Sine

**Affiliations:** 1 Departments of Physiology and Biomedical Engineering, Mayo Clinic College of Medicine and Science, Rochester, MN 55905, USA; 2 Departments of Molecular Pharmacology and Experimental Therapeutics, Mayo Clinic College of Medicine and Science, Rochester, MN 55905, USA; 3 Department of Neurology, Baylor College of Medicine, Houston, TX 77030, USA; 4 Department of Neuroscience, Baylor College of Medicine, Houston, TX 77030, USA; 5 Department of Molecular and Human Genetics, Baylor College of Medicine, Houston, TX 77030, USA; 6 Departments of Neurology, Mayo Clinic College of Medicine and Science, Rochester, MN 55905, USA

**Keywords:** sleep-related hyperkinetic epilepsy, neuronal nicotinic acetylcholine receptor, gain-of-function variant, patch-clamp, single ion-channel, ion-channel gating, spontaneous channel gating, ion-channel conductance

## Abstract

We describe genetic and molecular-level functional alterations in the α4β2 neuronal nicotinic acetylcholine receptor (nAChR) from a patient with sleep-related hyperkinetic epilepsy and a family history of epilepsy. Genetic sequencing revealed a heterozygous variant c.851C>G in the *CHRNA4* gene encoding the α4 subunit, resulting in the missense mutation p.Ser284Trp. Patch clamp recordings from genetically engineered nAChRs incorporating the α4-Ser284Trp subunit revealed aberrant channel openings in the absence of agonist and markedly prolonged openings in its presence. Measurements of single channel current amplitude distinguished two pentameric stoichiometries of the variant nAChR containing either two or three copies of the α4-Ser284Trp subunit, each exhibiting aberrant spontaneous and prolonged agonist-elicited channel openings. The α4-Ser284 residue is highly conserved and located within the M2 transmembrane α-helix that lines the ion channel. When mapped onto the receptor’s three-dimensional structure, the larger Trp substitution sterically clashes with the M2 α-helix from the neighboring subunit, promoting expansion of the pore and stabilizing the open relative to the closed conformation of the channel. Together, the clinical, genetic, functional, and structural observations demonstrate that α4-Ser284Trp enhances channel opening, predicting increased membrane excitability and a pathogenic seizure phenotype.

## 1. Introduction

Inherited forms of hyperkinetic epilepsy occurring during sleep have been associated with alterations in multiple genes, including *CHRNA4* and *CHRNB2* that encode subunits of the α4β2 nAChR [1,2,3]. Nocturnal seizures are very common clinically and may be therapeutically refractory, but only a minority have been linked to Mendelian causes. Pathogenic variants of the α4β2 nAChR described so far are heterozygous and dominantly transmitted with high penetrance. Accordingly, such disorders are classified as Sleep-related Hyperkinetic Epilepsy (SHE) [4], previously known as autosomal dominant nocturnal frontal lobe epilepsy (ADNFLE). α4β2 nAChRs distribute widely throughout the central nervous system where they localize at pre- and post-synaptic sites and enhance neurotransmitter release or mediate excitation, respectively [5,6]. They exist in two major pentameric assemblies that differ in subunit stoichiometry [7,8], agonist sensitivity [9], calcium modulation of channel gating [10], and sensitivity to allosteric modulators [11]. Previous work established that SHE variants in either the α4 or β2 subunits enhance sensitivity to agonist as demonstrated by electrophysiological recordings of macroscopic currents from genetically reconstituted nAChRs harboring the pathogenic variant subunit [12,13]. However, enhanced agonist sensitivity as assessed by macroscopic current recordings could arise through either enhanced agonist binding or enhanced channel gating [14]. In addition, recordings of macroscopic currents cannot distinguish changes in unitary current amplitude or changes that differ among stoichiometric forms of the receptor. Thus, elucidating pathogenic mechanisms at the molecular level requires functional measurements from individual receptor channels.

Herein we describe a patient with SHE and a family history of epilepsy, identify a heterozygous variant in the CHRNA4 gene, and genetically reconstitute receptors harboring the altered α4 subunit. We then use patch clamp electrophysiology to observe the function of individual receptors and demonstrate aberrant function of the two major stoichiometric forms of the α4β2 nAChR. Our functional measurements, together with the location of the altered residue within the three-dimensional structure of the α4β2 nAChR, show that the variant in the CHRNA4 gene produces a pathogenic gain of function via enhanced gating of the receptor channel.

## 2. Results

### 2.1. Clinical Evaluation

At the time of the initial clinical evaluation the patient was a 36-year-old right-handed male non-smoker with a history of sleep-related hyperkinetic seizures starting at age 5. The patient’s son and father’s cousin were reported to be diagnosed with childhood-onset epilepsy of unknown etiology but were not examined. The patient’s seizure semiology was described as arising from sleep with sudden onset multidirectional tongue movements, intermittent eye closure, and gagging vocalizations, followed by bilateral symmetric upward arm flexion and asynchronous leg movements. There was no loss of consciousness or bowel or bladder incontinence. Postictally, the patient could recall details of the event but was typically fatigued and drowsy and could resume sleep. These events typically occurred every 20–30 min overnight without medication and rarely secondarily generalized. Multiple medication trials including carbamazepine, clonazepam, lamotrigine, and zonisamide were unsuccessful in achieving seizure freedom. After admission to an Epilepsy Monitoring Unit and medication tapering, 89 behavioral events with the previously described seizure semiology were video recorded; however, the EEG in these events was obscured by myogenic artifact. In two such events, the typical semiology progressed to right arm extension, right-sided upper extremity clonic movement, and electroclinical evolution to a bilateral tonic-clonic seizure. However, there were no electrographic lateralizing features.

**Figure 1:**
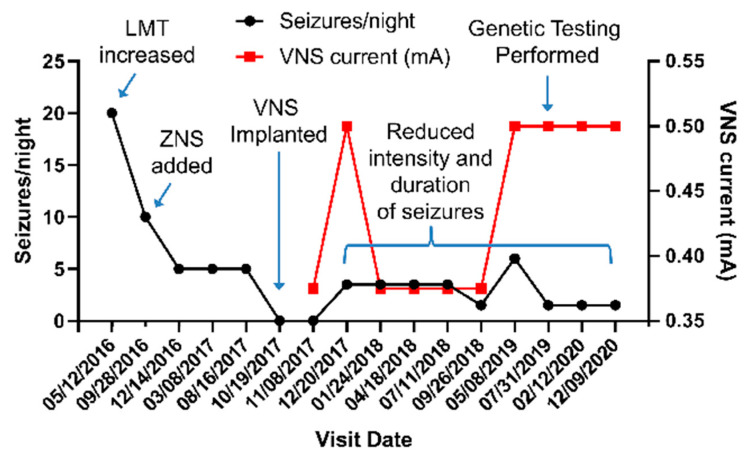
Patient Timeline. Seizure burden overall improved with increasing dose of lamotrigine (LMT), addition of zonisamide (ZNS), and titration of vagus nerve stimulator (VNS) settings.

**Figure 2:**
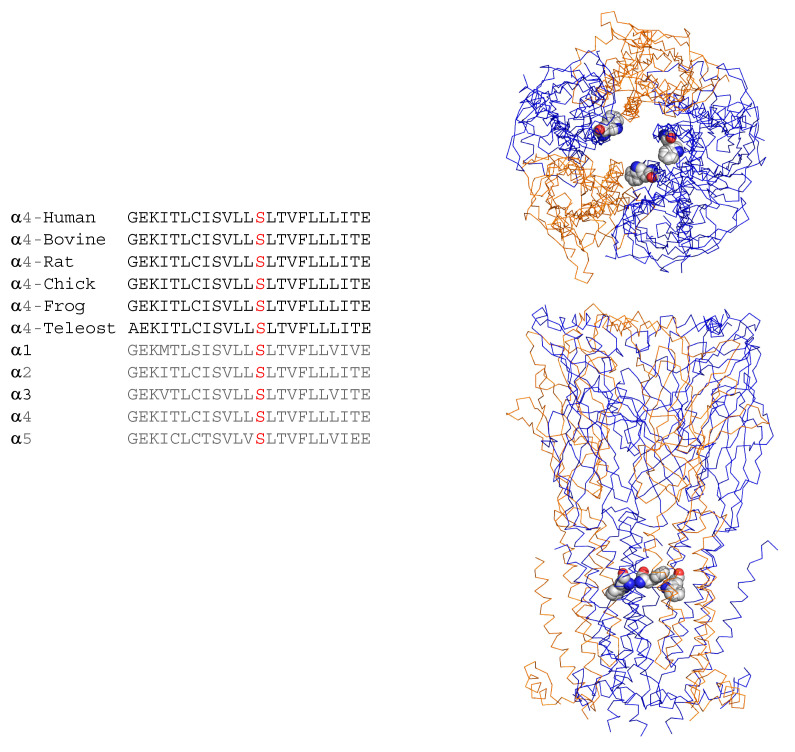
Sequence alignment and location of the α4-Ser284Trp residue in the structure of the α4β2 nAChR. *Upper panel*, sequence alignment of the M2 transmembrane domain of the α4 subunit for different vertebrate species and the indicated human α-subunit subtypes. The α4-Ser284 residue is highlighted in *red* font. *Lower panel*, top and side views of the structure of the (α4)_3_(β2)_2_ stoichiometric form of the receptor (PDB: 6CNK) with the α4 subunits in *blue*, the β2 subunits in *orange*, and the substituted Trp sidechain at position 284 in space filling representation.

The patient was evaluated for epilepsy surgery but was found to have normal brain magnetic resonance imaging (MRI), normal brain positron emission tomography (PET) scan, an inconclusive ictal single-photon emission computerized tomography (SPECT) scan, and a magnetoencephalogram (MEG) showing left greater than right bi-frontal epileptiform discharges. After discussion in a multidisciplinary patient management conference, a vagus nerve stimulator (VNS) was implanted. With medication changes and VNS implantation (Figure 1), the frequency, intensity and duration of seizures improved, but he continued to have 1–2 seizures/night despite maximal tolerable medication dosing and VNS management. Subsequent next-generation genetic sequencing (Invitae Epilepsy Panel, 146 genes) revealed the heterozygous variant c.851C>G in the *CHRNA4* gene resulting in the missense mutation p.Ser284Trp within the α4 subunit α4β2 nAChR, classified by the laboratory as a variant of uncertain significance. The α4-Ser284 residue is conserved among *CHRNA4* genes from vertebrate species, as well as human *CHRNA1* through *CHRNA7* genes (Figure 2). Located within the M2 transmembrane α-helix that lines the ion channel, α4-Ser284Trp maps onto the cryo-electron microscopic structure of the α4β2 nAChR [8] such that the large Trp sidechain impinges upon the M2 transmembrane α-helix from the neighboring subunit in a manner that would promote expansion of the pore (Figure 2).

### 2.2. Expression of nAChRs Harboring α4-Ser284Trp

**Figure 3:**
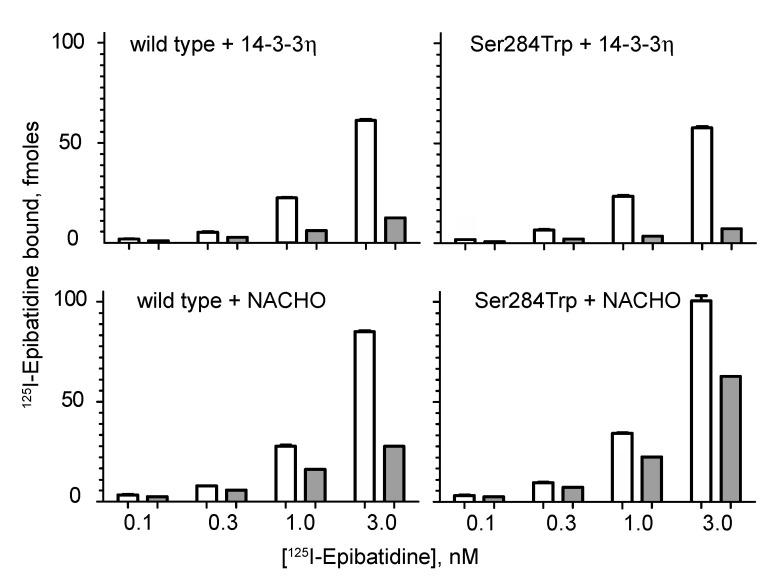
Expression of wild type and α4-Ser284Trp nAChRs in Bosc 23 cells. Cells were transfected with cDNAs encoding the wild type or mutant α4 subunit plus the β2 subunit and the indicated chaperone, either 14-3-3η or NACHO. Suspensions of intact cells were prepared, incubated with the indicated concentrations of ^125^I-epibatidine, and radio-ligand bound to the cells was determined as described in Materials and Methods. Open bars represent duplicate measurements of specific binding of ^125^I-epibatidine alone, whereas gray bars represent binding in the presence of 6 mM ACh. Each determination represents binding to one tenth of the cells harvested from a 10 cm tissue culture dish. Error bars larger than a line width indicating the SD are shown.

To evaluate the ability of the α4-Ser284Trp subunit to incorporate into cell surface receptors, we engineered the Ser284Trp variant into the cDNA encoding the human α4 subunit that was subcloned into a mammalian expression vector, co-expressed the α4 and β2 subunit cDNAs in Bosc-23 cells, a human fibroblast cell line derived from 293 HEK cells, and measured binding of radio-labeled epibatidine to intact cells. Given that epibatidine is membrane permeable, to determine the fraction of labeled receptors on the cell surface, parallel measurements were conducted in the presence of a high concentration of ACh, which contains a quaternary ammonium moiety that renders it membrane impermeable. To bias expression toward the (α4)_3_(β2)_2_ stoichiometry, the chaperone 14-3-3η was co-transfected along with α4 and β2 subunits, whereas to bias expression toward the (α4)_2_(β2)_3_ stoichiometry the chaperone NACHO was co-transfected [15]. Non-specific binding was determined by parallel measurements conducted on cells transfected with the β2 subunit alone. Broadly, robust binding of radio-labeled epibatidine was observed over the nanomolar range of concentrations tested, and a substantial portion of the total binding was inhibited by ACh (Figure 3). For cells co-transfected with 14-3-3η, a majority of the total epibatidine binding was inhibited by ACh for both wild type and α4-Ser284Trp receptors, indicating efficient trafficking of the (α4)_3_(β2)_2_ stoichiometry to the cell surface. For cells co-transfected with NACHO, a smaller yet substantial fraction of the total binding was inhibited by ACh, indicating somewhat less efficient trafficking of the (α4)_2_(β2)_3_ stoichiometry to the cell surface. Thus, receptors containing either wild type or variant Ser284Trp α4 subunits exhibit robust amounts of radio-labeled epibatidine binding, showing that the α4-Ser284Trp subunit is expression competent and providing a foundation for patch clamp studies.

### 2.3. Patch Clamp Studies of nAChRs Harboring α4-Ser284Trp

**Figure 4:**
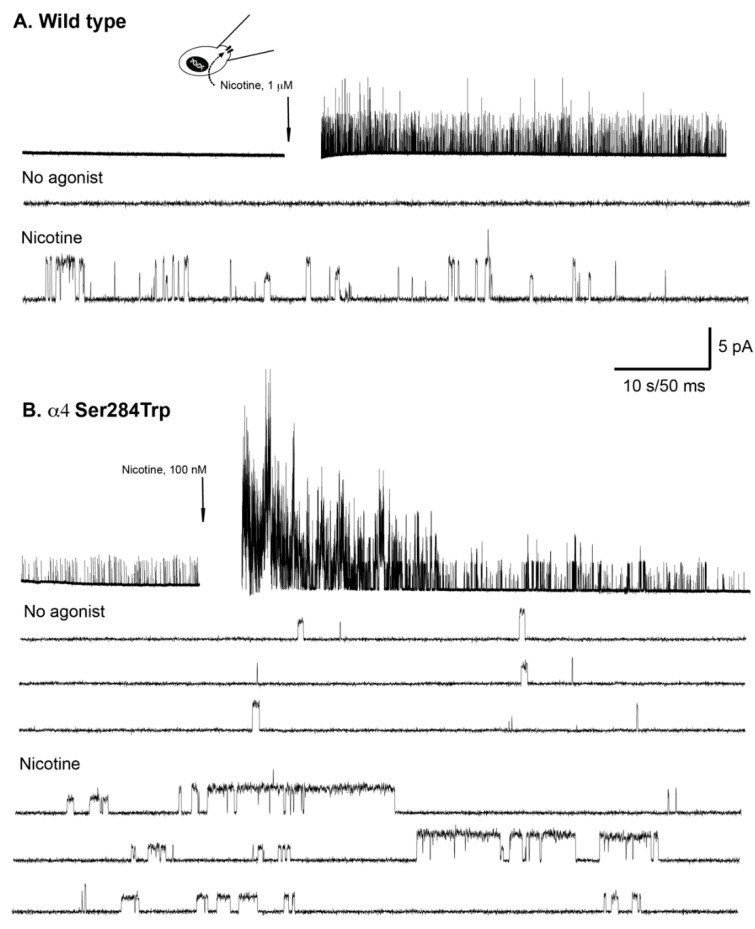
Single channel recordings from wild type and α4-Ser284Trp nAChRs before and after addition of nicotine. (**A**) Recording from a Bosc-23 cell expressing wild type α4β2 nAChRs. *Upper trace* shows current recorded from the same patch of membrane before and after addition of 1 µM nicotine to the extracellular solution (1 kHz Gaussian filter). Noise artifact during addition of nicotine is removed (gap). *Lower traces* show segments of the upper trace with increased time resolution (3 kHz Gaussian filter). (**B**) Recording from a Bosc-23 cell expressing α4Ser284Trpβ2 nAChRs. *Upper trace* shows current recorded from the same patch of membrane before and after addition of 100 nM nicotine to the extracellular solution (1 kHz Gaussian filter). *Lower traces* show segments of the upper trace with increased time resolution (3 kHz Gaussian filter). The recordings in A and B were obtained in the cell-attached patch configuration at a membrane potential of −70 mV.

To assess the functional consequences of the α4-Ser284Trp variant, we monitored activation of receptors at the single channel level using the patch clamp technique. Initially we made patch clamp recordings before and after application of nicotine, a membrane permeable agonist, to receptors within the same patch of cell membrane. For any new type of nAChR, recordings before and after agonist application are necessary to demonstrate that the observed channel openings represent the activity of authentic nicotinic receptors. An exemplar experiment from three independent experiments that gave the same qualitative results is illustrated in Figure 4. For the wild type nAChR in the absence of nicotine no single channel activity was observed, whereas addition of nicotine to the external bathing solution elicited robust channel openings with either small or large current amplitude (Figure 4A); previous work showed that channel openings with small current amplitude correspond to the (α4)_2_(β2)_3_ stoichiometry and openings with large amplitude to the (α4)_3_(β2)_2_ stoichiometry [7]. By contrast, nAChRs harboring the α4-Ser284Trp variant exhibited channel openings with both small and large current amplitude in the absence of nicotine, while addition of nicotine markedly increased the incidence and the durations of channel openings with small and large current amplitude (Figure 4B). Thus, the α4-Ser284Trp variant promotes spontaneous channel opening of the two major stoichiometric forms of the α4β2 nAChR, predicting enhanced excitability even in the absence of cholinergic transmission. In addition, α4-Ser284Trp prolongs the durations of agonist-elicited channel openings, predicting increased excitation during cholinergic transmission.

**Figure 5:**
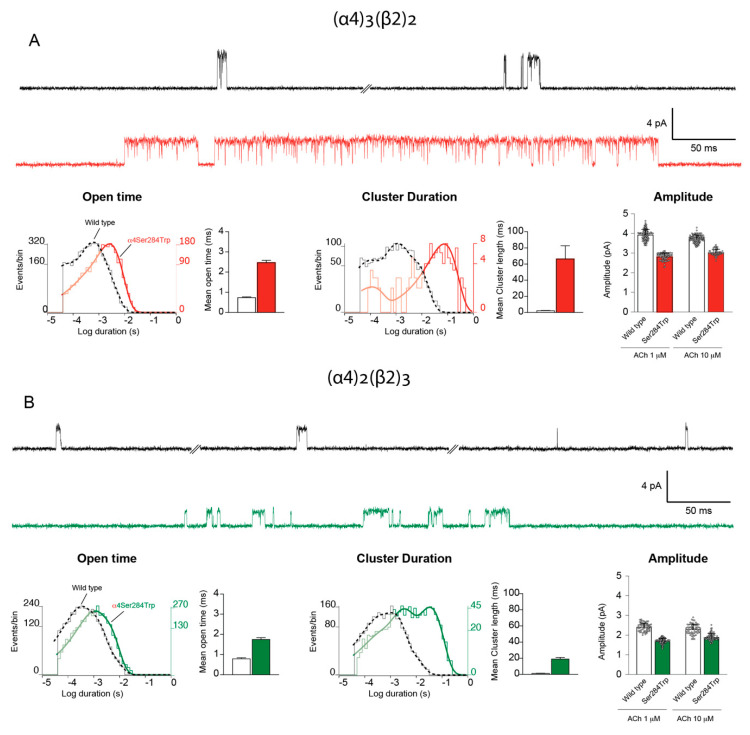
Single channel recordings from wild type and α4Ser284Trpβ2 nAChRs activated by ACh. (**A**) Recording from a Bosc−23 cell expressing the (α4)3(β2)2 stoichiometry of wild type (black trace) or mutant (red trace) nAChRs in the presence of 10 µM ACh. Corresponding histograms of open and cluster durations, fitted by the sum of exponentials, are shown below. Unitary current amplitude for wild type and mutant nAChRs is compared for recordings in the presence of either 1 or 10 µM ACh. Each symbol represents the open channel current from a continuous data segment containing multiple channel openings. (**B**) Recording from a Bosc-23 cell expressing the (α4)2(β2)3 stoichiometry of wild type (black trace) or α4-Ser284Trp (green trace) nAChRs in the presence of 1 µM ACh. Corresponding histograms of open and cluster durations, fitted by the sum of exponentials, are shown below. Unitary current amplitude for wild type and α4-Ser284Trp nAChRs is compared for recordings in the presence of either 1 or 10 µM ACh, as in panel A. The recordings in A and B were obtained in the cell-attached patch configuration at a membrane potential of −70 mV. The bandwidth for display is 5 kHz.

To quantify the functional consequences of the α4-Ser284Trp variant, we recorded single channel currents through each stoichiometric form of the receptor in the presence of the physiological neurotransmitter ACh. In these studies recordings were made with a specified concentration of ACh in the solution within the patch clamp recording pipette. To generate a receptor population enriched in one or the other stoichiometric form, we co-expressed the α4 and β2 subunit cDNAs together with a cDNA encoding either NACHO or 14-3-3η (Materials and Methods). For the (α4)_3_(β2)_2_ stoichiometry, α4-Ser284Trp markedly prolonged ACh-elicited channel openings, and the mean duration of channel openings was 3.3–4.8-fold greater than the wild-type counterpart (Figure 5A; Table 1). The α4-Ser284Trp variant also promoted clustering of successive openings by the same nAChR channel. To quantify the durations of clusters, we defined a cluster as a series of channel openings flanked by closings briefer than a specified closed time; this closed time was determined from the histogram of closed dwell times as the point of intersection between the closed time component with longest mean duration and the succeeding briefer component (Materials and Methods). The analysis revealed that the α4-Ser284Trp variant increased the mean duration of clusters for the (α4)_3_(β2)_2_ stoichiometry by 20- to 28-fold compared to wild type (Figure 5A; Table 1). Thus, the α4-Ser284Trp variant prolongs ACh-elicited channel openings, and upon channel closing, it also promotes return to the open state rapidly and with increased probability. Analysis of the unitary current amplitude revealed that the α4-Ser284Trp variant reduced the mean amplitude from an average of 3.8 to 2.9 pA at a reference membrane potential of −70 mV (Table 2), a reduction of ~24%.

For the (α4)_2_(β2)_3_ stoichiometry, the α4-Ser284Trp variant also prolonged ACh-elicited channel openings and clusters of channel openings (Figure 5B), but the effects were smaller than observed for the (α4)_3_(β2)_2_ stoichiometry; the mean channel open duration increased by 2.2-fold and the mean cluster duration increased by 14-fold (Figure 5B; Table 1). The α4-Ser284Trp variant also reduced the mean unitary current amplitude from an average of 2.35 to 1.8 pA at the reference membrane potential of −70 mV (Table 2), a reduction of ~23%. In comparing the two stoichiometric forms, the α4-Ser284Trp variant increased the mean open and cluster durations approximately in proportion to the number of α4-Ser284Trp subunits, whereas the reduction in the unitary current amplitude did not depend on the number of α4-Ser284Trp subunits. Thus, recordings of ACh-elicited channel openings by nAChRs harboring the α4-Ser284Trp variant reveal a marked increase in the durations of channel opening episodes of both stoichiometric forms and a smaller decrease in the unitary current amplitude of each form.

To quantify the effects of the α4-Ser284Trp variant on channel openings elicited by a chemically different type of agonist, we recorded channel openings elicited by nicotine, which contains a tertiary rather than a quaternary amine group; for these studies’ nicotine was added to the external solution using the method described in Figure 4. For the (α4)_3_(β2)_2_ stoichiometry, the α4-Ser284Trp variant increased the mean duration of nicotine-elicited channel openings by 4.9-fold and increased the mean cluster duration by 21-fold (Table 1); both increases are very close to those observed with ACh as the agonist (Table 1). However, for the (α4)_2_(β2)_3_ stoichiometry, the α4-Ser284Trp variant increased the mean duration of nicotine-elicited channel openings by only 1.2-fold and increased the mean cluster duration by 3.4-fold (Table 1); both increases are smaller than observed with ACh, indicating the kinetic consequences of α4-Ser284Trp depend on the stoichiometric form of the α4β2 nAChR as well as the type of agonist. Comparison of the unitary current amplitudes of nicotine- versus ACh-elicited channel openings revealed virtually identical amplitudes for either wild type or α4-Ser284Trp nAChRs at a reference membrane potential of −70 mV (Table 2).

Recordings from the same patch of membrane, before and after nicotine application, allowed comparison of the mean open durations and current amplitudes of spontaneous and nicotine-elicited channel openings from nAChRs containing the α4-Ser284Trp variant. For the (α4)_3_(β2)_2_ stoichiometry, nicotine-elicited channel openings were 3-fold longer than spontaneous channel openings, while for the (α4)_2_(β2)_3_ stoichiometry, nicotine-elicited channel openings were 2-fold longer than spontaneous openings (Table 3). Thus, compared to channel openings in the absence of agonist, nicotine increased the mean open duration in proportion to the number of α4-Ser284Trp subunits. Measurements of unitary current amplitude showed that the α4-Ser284Trp variant reduced the amplitudes of both spontaneous and nicotine-elicited channel openings for each stoichiometric form, but that the current amplitudes of the openings were indistinguishable in the absence or presence of nicotine (Table 4). 

## 3. Discussion

We describe the clinical profile and the genetic and molecular-level functional alterations in a neuronal nAChR from a patient with SHE since childhood and a family history of epilepsy. Genetic sequencing revealed the heterozygous variant c.851C>G in the *CHRNA4* gene, resulting in a change in the protein coding sequence of the α4 subunit, p.Ser284Trp. Following genetic reconstitution of α4β2 nAChRs harboring the α4-Ser284Trp subunit, we documented the functional consequences of the genetic variant at the single channel level. The α4-Ser284Trp variant and a second variant α4-Ser284Leu were previously reported as potentially pathogenic based on clinical seizure phenotype and detection of the variants in affected relatives [16]. Although functional consequences of the α4-Ser284Leu variant have been investigated using whole cell patch clamp techniques [17], the functional consequences of the α4-Ser284Trp variant have not been investigated by either whole cell or single channel patch clamp techniques. Studies of genetic variants causing congenital myasthenic syndromes have benefitted greatly from analyses of muscle AChRs using single channel patch clamp techniques, revealing aberrations in elementary functional steps in the activation process [18,19], however single channel studies of genetic variants in nAChRs from the CNS have been limited. Our functional studies at the single channel level reveal multifaceted functional alterations in each of the two stoichiometric forms of the pentameric α4β2 nAChR, including channel opening in the absence of agonist, prolonged agonist-elicited channel openings, enhanced clustering of agonist-elicited channel openings, reduction of the single channel current amplitude, and changes in the mean channel open time that depend on the stoichiometric form and type of agonist. The functional consequences of the α4-Ser284Trp variant predict increased neuronal excitability in the absence of cholinergic signaling and a further increase during signaling.

Fundamental processes in nAChR function include agonist binding, channel gating, ion permeation, and desensitization. Previous work on SHE genetic variants demonstrated enhanced agonist sensitivity in dose-response measurements of whole cell macroscopic currents from genetically reconstituted nAChRs [12,13,17,20,21]. However, enhanced agonist sensitivity, as measured by whole cell macroscopic currents, could arise from either enhanced agonist binding or enhanced channel gating [14]. Our single channel measurements demonstrate that the α4-Ser284Trp variant enhances channel gating, which arises through increased stability of the open channel state and enhanced reopening of the channel after closing. The net effect of α4-Ser284Trp is up to a 20-fold increase in the duration of channel opening episodes in response to agonist. During synaptic transmission, the increased durations of channel opening episodes would produce a commensurate increase in the duration of excitation. A more subtle effect of α4-Ser284Trp is the occurrence of channel opening in the absence of agonist, a phenomenon not observed with the wild type α4β2 nAChR. Opening of the channel in the absence of agonist would produce tonic depolarization and consequently a reduction in the threshold for excitation by voltage-gated ion channels linked to action potential firing and neurotransmitter release. The α4-Ser284Trp variant also reduces the rate of ion permeation during channel openings, but the reduction is modest at ~24%. Such a reduction could arise via steric occlusion of the pore owing to the bulky Trp substitution, which could also alter the efficacy of channel blocking drugs considered as possible therapeutics. A previous study of the α4-Ser284Leu variant demonstrated enhanced desensitization [22], which would partially counter the gain of function during prolonged exposure to ACh as occurs during paracrine signaling [23,24]. Overall, our single channel analyses reveal pleotropic changes in nAChRs containing α4-Ser284Trp that culminate in a pronounced gain of function that likely contributes to the patient’s epilepsy phenotype.

The α4β2 nAChR is a pentamer that assembles in two major stoichiometric forms, (α4)_3_(β2)_2_ and (α4)_2_(β2)_3_ [8], which are readily distinguished by their unitary current amplitude [7,10]. Our results demonstrate that the α4-Ser284Trp subunit incorporates into both stoichiometric forms, and that with ACh as the agonist, the mean duration of channel opening episodes is prolonged in proportion to the number of α4-Ser284Trp subunits. However, with nicotine as the agonist the increase in the mean duration of channel opening episodes differs between the two stoichiometric forms, with the (α4)_3_(β2)_2_ stoichiometry exhibiting a much larger increase than the (α4)_2_(β2)_3_ stoichiometry. In laboratory animals the two stoichiometric forms have been detected by photobleaching of fluorescent α4β2 nAChRs, revealing roughly equal proportions of each stoichiometric form in most brain regions, but that the proportions change upon nicotine treatment [25]. In patients with ADNFLE, PET imaging using a high affinity ^18^F-labeled agonist for α4β2 nAChRs showed increased labeling in mesencephalic regions and reduced labeling in the prefrontal cortex compared to controls [26]. Thus, aberrant receptor function on the time scale of synaptic transmission may promote longer term changes in the density, distribution, and stoichiometric forms of the α4β2 nAChR that impact the pathophysiology. However, on a longer time scale, as occurs with intake of nicotine, the effects of enhanced channel gating may preferentially impact one stoichiometric form over the other.

An additional consideration follows from the heterozygous genotype coupled with the presence of multiple α4 subunits in the pentameric α4β2 nAChR. With a heterozygous genotype, and random incorporation of wild type and variant subunits, individual receptors with different numbers of α4-Ser284Trp subunits would be expected- three for the (α4)_2_(β2)_3_ form and seven for the (α4)_3_(β2)_2_ form. That wild type and variant subunits incorporate randomly is plausible given our measurements of radio-labeled epibatidine binding showing that both wild type and α4-Ser284Trp subunits enable robust expression of cell surface nAChRs. Among the multiple possible molecular forms of the variant nAChR, our patch clamp studies document aberrant function of two of these forms, those with either two or three copies of the α4-Ser284Trp subunit. The observation that the functional aberration accrues in proportion to the number of α4-Ser284Trp subunits predicts that molecular forms containing both variant and wild type subunits would exhibit smaller enhancements of channel gating than observed herein; thus, molecular forms containing solely the α4-Ser284Trp variant represent upper bounds in the degree of pathogenicity.

The five subunits of the nAChR align as staves of a barrel to form a central channel through which ions flow. Each subunit is situated with one of its four transmembrane α-helices, known as M2, contributing to the wall of the channel. By convention, the residues of M2 are numbered beginning with the −1′ residue that forms the ion selectivity filter at the intracellular end and the 20′ residue at the extracellular end. The altered α4-Ser284 residue from the patient occupies the 10′ position of M2 and thus is located three helical turns above the ion selectivity filter. In the resting state the M2 α-helices from each subunit pack against the central axis preventing ion flow, but in the open state the M2 α-helices move away from the central axis such that the distances between neighboring M2 α-helices increase [27]. For receptors containing the α4-Ser284Trp variant, the increased bulk of the Trp sidechain may facilitate the increase in inter-helical distance and thus favor the open over the closed state of the channel. By biasing toward the open state, the variant would increase the probability of channel opening in the absence of agonist and increase the durations of agonist-elicited channel openings. In addition, the bulky Trp side chain may narrow the cross section of the channel in the open state and account for the decrease in unitary current amplitude. Thus, the functional consequences of the α4-Ser284Trp variant highlight the crucial role of the fine structure of M2 in tuning both the stability of the open state as well as the rate of ion flow through the channel.

Elucidating the deleterious functional consequences of variants in genes encoding the α4β2 nAChR has implications for developing targeted therapeutics. A non-competitive channel blocker would be expected to counter the gain of function due to enhanced channel opening. For the patient variant described herein the bulky Trp substitution, which reduces the unitary current amplitude, may create a drug binding site selective for the variant over the wild type nAChR. In addition, given that expression of the α4β2 nAChR can change in the presence of nicotine or in patients with SHE, chronic drug treatment prior to the onset of symptoms may be beneficial in preventing plastic changes in response to gain of function genetic variants of the nAChR. Our overall findings provide a mechanistic context in which to develop therapeutics to neutralize the effects of the α4-Ser284Trp variant and offer a paradigm to delineate molecular level functional abnormalities in patients with genetic epilepsy.

## 4. Materials and Methods

### 4.1. Expression of Human (α4)_3_(β2)_2_ and Human (α4)_2_(β2)_3_ AChRs

cDNAs encoding human α4 and β2 subunits, and the chaperone proteins 14-3-3η and NACHO were individually sub-cloned into mammalian expression vectors, either pCI (Promega, Madison, WI, USA) or pRBG4, as previously described [15]. The patient variant α4-Ser284Trp was generated using the QuickChange site-directed mutagenesis kit (Agilent Technologies, Palo Alto, CA, USA) and was confirmed by sequencing; residue numbering of the α4 subunit begins with the first amino acid of the signal peptide that is removed in the final protein. Co-transfection with either 14-3-3η or NACHO allowed preferential expression of (α4)_3_(β2)_2_ or (α4)_2_(β2)_3_ AChRs, respectively. Bosc-23 cells [28], a cell line derived from HEK 293 cells, were maintained in Dulbecco’s modified Eagle’s medium (DMEM, Gibco, Grand Island, NY, USA) containing 10% fetal bovine serum, and transfected by calcium phosphate precipitation, as previously described [29]. To bias the stoichiometry towards (α4)_3_(β2)_2_, cells were transfected with a 10:1:10 ratio of α4, β2, and 14-3-3η cDNAs. The amounts of transfected α4 and β2 cDNAs were 6 and 0.6 μg for each 35 mm culture dish of cells. To bias the stoichiometry towards (α4)_2_(β2)_3_, cells were transfected with a 1:1:0.3 ratio of α4, β2, and NACHO cDNAs. The amounts of transfected α4 and β2 cDNAs were 3 and 3 μg for each 35 mm culture dish of cells. A cDNA encoding green fluorescent protein was included in all transfections. Transfections were carried out for 4 to 6 h, followed by medium exchange. Single channel recordings were made 48–72 h post-transfection.

### 4.2. Drugs

Acetylcholine chloride and nicotine were purchased from Sigma-Aldrich (St. Louis, MO, USA).

### 4.3. Radio-Ligand Binding

To measure expression of the α4β2 nAChR, transfected cells from two 10 cm tissue culture dishes were harvested by gentle agitation in phosphate buffered saline, centrifuged at 2500 rpm for 1 min and resuspended in (in mM): 140 KCl, 5.4 NaCl, 1.8 CaCl_2_, 1.7 MgCl_2_ and 10 HEPES, with the pH adjusted to 7.4 with NaOH. Cell suspensions were divided into equal aliquots, which were incubated with specified concentrations of ^125^I-epibatidine (kindly provided by Dr. Vanda Lennon, Neuroimmunology Laboratory, Mayo Clinic) for one hour at 21 °C with or without preincubation with 6 mM acetylcholine chloride. Cell suspensions were filtered using a Brandel M-48T cell harvester (Gaithersburg, MD, USA) and radio-ligand bound to the cells was measured using a γ-counter. To determine non-specific binding, identical procedures were applied to cells transfected with cDNA encoding the β2 subunit.

### 4.4. Single Channel Recordings

Patch clamp recordings were obtained from Bosc-23 cells expressing α4β2 nAChRs using the cell-attached patch configuration at a temperature of 21 °C and membrane potential of −70 mV. Patch pipettes were fabricated from glass capillary tubes (7052, King Precision Glass, Claremont, CA, USA), coated with Sylgard (Dow Corning, Midland, MI, USA) and heat polished to a resistance of 5–10 mega-ohms. The bath and pipette solutions contained (in mM): 140 KCl, 5.4 NaCl, 1.8 CaCl_2_, 1.7 MgCl_2_ and 10 HEPES, with the pH adjusted to 7.4 with KOH. Concentrated stock solutions of nicotine and ACh (Sigma-Aldrich) were stored at −80 °C and diluted to the final concentrations in pipette solution on the day of each experiment. Single-channel currents were recorded using an Axopatch 200B patch-clamp amplifier (Molecular Devices, San Jose, CA, USA) with a gain of 100 mV/pA and the internal Bessel filter at 100 kHz. Upon formation of a giga-ohm seal a constant command voltage was applied to the interior of the patch pipette to establish the membrane potential. Currents were digitized at 20 µs intervals using a National Instruments model BNC-2090 A/D converter with a PCI 6111e acquisition card and recorded to the hard disk of a PC using the program Acquire (Bruxton Corporation, Seattle WA, USA). For analysis the acquired data were filtered at a bandwidth of 5 kHz using a Gaussian filter.

Analysis of single channel currents was performed using the program TAC 4.3.3 (Bruxton). To analyze open and closed dwell times, single channel events were detected using the half amplitude threshold criteria, dwell times were plotted using a logarithmic abscissa and a square root ordinate with an imposed dead time of 30 µs, and the sum of exponentials was fitted to the histograms by maximizing the likelihood. To analyze single channel current amplitude, an all-points histogram was generated for a sweep of data containing multiple channel openings, and a Gaussian function was fitted to the baseline and open channel current levels. The difference between the means of the baseline and open channel currents was averaged for five to ten sweeps to yield the mean current amplitude for a given experimental condition, as described [7].

To quantify the durations of clusters of channel openings, a cluster was defined as a series of openings interspersed by closings briefer than a specified critical duration (τ_crit_). This duration was determined from the histogram of closed dwell times as the point of intersection between the exponential component of closings with longest mean duration and that of the preceding briefer component and ranged between 1 and 3 ms.

### 4.5. Statistics

For statistical analysis, data are presented as the mean and S.D. Statistical comparisons were carried out using an unpaired non-parametric *t*-test using GraphPad Prism 9.2.0 (GraphPad Software Inc., San Diego, CA, USA). Differences between the mean values of measurements being compared were considered significant if the *p*-value was less than 0.01.

## Figures and Tables

**Table 1:** Effect of α4Ser284Trp on mean open time and mean cluster duration.

Receptor-Type (Number of Patches)	Agonist, µM	Mean Open Time (ms) 95% CI)	Fold Change Relative to Wild Type	Mean Cluster Duration (ms) (95% CI)	Fold Change Relative to Wild Type	Statistical Difference between Wild Type and α4-Ser284Trp
(α4)3(β2)2 (*n* = 9)	ACh, 1	0.66 (0.63–0.68)	4.8	1.31 (1.23–1.40)	20.3	Yes
(α4S284W)3(β2)2 (*n* = 7)	ACh, 1	3.18 (3.01–3.35)		26.6 (22.8–33.3)		
(α4)3(β2)2 (*n* = 14)	ACh, 10	0.75 (0.72–0.78)	3.3	2.36 (2.18–2.54)	28.1	Yes
(α4S284W)3(β2)2 (*n* = 5)	ACh, 10	2.48 (2.37–2.58)		66.4 (50.1–82.7)		
(α4)2(β2)3 (*n* = 5)	ACh, 1	0.80 (0.76–0.84)	2.2	1.35 (1.26–1.44)	14.2	Yes
(α4S284W)2(β2)3 (*n* = 7)	ACh, 1	1.76 (1.68–1.83)		19.2 (17.4–21.0)		
(α4)2(β2)3 (*n* = 5)	ACh, 10	0.77 (0.74–0.79)	2.2	1.49 (1.40–1.59)	13.5	Yes
(α4S284W)2(β2)3 (*n* = 7)	ACh, 10	1.66 (1.58–1.74)		20.1 (18.1–23.1)		
(α4)3(β2)2 (*n* = 3)	Nicotine, 1	0.90 (0.87–0.93)	4.9	1.65 (1.57–1.73)	20.7	Yes
(α4S284W)3(β2)2 (*n* = 3)	Nicotine, 1	4.45 (4.21–4.68)		34.3 (28.9–39.7)		
(α4)2(β2)3 (*n* = 3)	Nicotine, 1	1.17 (1.11–1.23)	1.21	1.42 (1.34–1.50)	3.4	Yes
(α4S284W)2(β2)3 (*n* = 3)	Nicotine, 1	1.41 (1.37–1.45)		4.79 (4.50–5.08)		

**Table 2:** Effect of α4Ser284Trp on single channel current amplitude.

Receptor-Type (Number of Patches)	Agonist, µM	Current Amplitude (pA) at −70 mV Mean (95% CI); N = Number of Openings	Statistical Difference between Wild Type and α4-Ser284Trp
(α4)3(β2)2 (*n* = 9)	ACh, 1	3.9 (3.9–4); N = 120	Yes
(α4S284W)3(β2)2 (*n* = 8)	ACh, 1	2.8 (2.8–2.9); N = 70	
(α4)3(β2)2 (*n* = 11)	ACh, 10	3.7 (3.7–3.8); N = 112	Yes
(α4S284W)3(β2)2 (*n* = 5)	ACh, 10	3.0 (3.0–3.1); N = 54	
(α4)2(β2)3 (*n* = 5)	ACh, 1	2.4 (2.36–2.46); N = 60	Yes
(α4S284W)2(β2)3 (*n* = 7)	ACh, 1	1.7 (1.66–1.72); N = 81	
(α4)2(β2)3 (*n* = 5)	ACh, 10	2.3 (2.24–2.37); N = 60	Yes
(α4S284W)2(β2)3 (*n* = 7)	ACh, 10	1.9 (1.86–1.95); N = 74	
(α4)3(β2)2 (*n* = 3)	Nicotine, 1	3.9 (3.82–3.94); N = 74	Yes
(α4S284W)3(β2)2 (*n* = 3)	Nicotine, 1	2.9 (2.94–3.02); N = 74	
(α4)2(β2)3 (*n* = 3)	Nicotine, 1	2.7 (2.63–2.7); N = 95	Yes
(α4S284W)2(β2)3(*n* = 3)	Nicotine, 1	1.9 (1.84–1.88); N = 94	

**Table 3:** Effect of α4Ser284Trp on mean open times of spontaneous and Nicotine activated channel openings.

Receptor-Type (Number of Patches)	[Nicotine] µM	Mean open Time (ms) (95% CI)	Statistical Difference between Nicotine Activated and Spontaneous	Fold Change for Nicotine Activated Relative to Spontaneous
(α4S284W)3(β2)2 (*n* = 3)	0	1.54 (1.38–1.69)	Yes	2.9
(α4S284W)3(β2)2 (*n* = 3)	1	4.45 (4.21–4.68)		
(α4S284W)2(β2)3 (*n* = 3)	0	0.78 (0.66–0.90)	Yes	1.8
(α4S284W)2(β2)3 (*n* = 3)	1	1.41 (1.37–1.45)		

**Table 4:** Effect of α4Ser284Trp on amplitudes of spontaneous and Nicotine activated channel openings.

Receptor-Type (Number of Patches)	[Nicotine] µM	Current Amplitude (pA) at −70 mV Mean (95% CI); N = Number of Openings	Statistical Difference between Spontaneous and Nicotine Activated
(α4S284W)3(β2)2 (*n* = 3)	1	2.9 (2.94–3.02); N = 74	No
(α4S284W)3(β2)2 (*n* = 4)	0	2.9 (2.87–3.02); N = 68	
(α4S284W)2(β2)3 (*n* = 3)	1	1.9 (1.84–1.88); N = 94	No
(α4S284W)2(β2)3 (*n* = 5)	0	1.8 (1.75–1.89); N = 42	

## Data Availability

The data that support the findings of this study are available from the corresponding author upon reasonable request. Some data may not be made available because of privacy or ethical restrictions.
